# Editorial: Circadian rhythms of mental health

**DOI:** 10.3389/fnetp.2023.1279911

**Published:** 2023-10-24

**Authors:** Kneginja Richter, Thomas Penzel

**Affiliations:** ^1^ CuraMed Tagesklinik Nürnberg GmbH, Nuremberg, Germany; ^2^ University Hospital for Psychiatry and Psychotherapy, Paracelsus Medical University Nuremberg, Nuremberg, Germany; ^3^ Technische Hochschule Nürnberg Georg Simon Ohm, Nuremberg, Germany; ^4^ Interdisciplinary Sleep Medicine Centre, The Charité University Hospital, Berlin, Germany

**Keywords:** network physiology, circadian, chronobiology, mental health, psychiatry, psychology

## 1 Introduction

According to the WHO, there is no health without mental health. At the same time, however, there is no mental health without a healthy synchronization with the rhythm of life as expressed through the sleep-wake rhythm. Mental and physical health are always associated with changing of the physiologic States from Integrated Organ Network Interactions (Ivanov et al., 2021b).

The circadian Rhythm of the human organism is essential for an integrated network, with multi-component physiological systems, each with its own regulatory mechanism, continuously interact to coordinate their functions. Coordinated network interactions among organs according to the sleep-wake circadian Rhythm are essential to generating distinct physiological states and maintaining mental health (Ivanov et al., 2016; Ivanov et al., 2019).

The goal of this Research Topic was to provide scientific expertise on the impact of chronobiology and sleep on some important aspects of mental health such as psychopathology, suicidal behavior, violence, cognition, verbal emotional expression, personality traits, and the ability to make the right decisions.

In this collection of scientific papers, we focus on the strong relationship between physical and mental health with a special consideration of circadian and sleep-wake rhythms in humans. A short summary of the contributions included is presented here.

## 2 Sleep and sports: cognitive function in soccer athletes determined by sleep disruption and self-reported health, but not by decision-reinvestment

Pourhassan et al. used fit-trackers and psychometric questionnaires to assess sleep quality, wellbeing, and pain as well as decision reinvestment strategies in football players. Being a professional athlete requires the cognitive and mental ability to recall motoric movements stored in memory, to have a selective attention to observe other football players and their movements, and to make decisions very quickly during the match. The results of the study show that those football players who have interrupted sleep are also slower in recalling memory, which can be decisive for the strategy and outcome of the match. These findings confirm the need and importance to implement individual sleep coaching for football players.

## 3 The Mind after Midnight: nocturnal wakefulness, behavioral dysregulation, and psychopathology

Tubbs et al. propose the *Mind after Midnight* hypothesis positing that attentional biases, negative affect, altered reward processing, and prefrontal disinhibition interact to promote behavioral dysregulation and psychiatric disorders. This indicates that disrupted sleep is associated with significant nocturnal wakefulness leading to cognitive and behavioral dysregulation. The present review summarizes the evidence for day-night alterations in maladaptive behaviors, including suicide, violent crime, and substance abuse, and examines how mood, reward processing, and executive function differ during nocturnal wakefulness.

The Morning Twilight is associated with an elevated risk of suicide that is even three-fold higher between midnight and 6 a.m. than at any other time of the day after adjusting for population sleep/wake timing. The biological explanation for this psychopathological phenomenon is underlined by the fact that during nocturnal wakefulness, there is increased dopaminergic activation. This may adversely affect psychiatric symptoms associated with dopaminergic dysregulation. Secondly, nocturnal wakefulness may also produce a stress response with a surge in adrenergic signaling that further weakens prefrontal cortical activity and increases reflexive, impulsive decision such as suicidal behavior or violence.

Naturally, the *Mind after Midnight* can be very useful for personalized clinical applications involving interventions for the treatment of insomnia, pain, cravings, and other causes of nighttime awakening.

## 4 Big Five personality dimensions, chronotype, and DSM-V personality disorders

Staller and Randler work focuses on the highly interesting association between eveningness as a chonobiological variable and the items of the Big Five brief version. Their study on 630 persons proved a relationship between eveningness and DSM-5-personality traits, (evening-oriented participants showing a higher PID-5 score: morningness −0.208/*p* < 0.001; eveningness: 0.153/*p* < 0.001). These results suggest that evening-types are prone to PDs, regardless of the construct used (MESSi/midpoint of sleep). The authors could thus confirm the hypothesis that eveningness is related to a high risk of PDs. Even more interestingly, eveningness was described as a psychological facet of the chronobiological profile of the participants. These findings could open up the possibility to predict PD early in life and to therefore initiate some measures for prevention.

## 5 Depressive symptoms, circadian strain, light exposure, in rural communities of southern Brazil

In their paper, Pilz et al. a research on the impact of social jetlag and light exposure on depressive Symptoms in Quilombolas communities in Southern Brazil using the Munich ChronoType Questionnaire (MCTQ), the Beck Depression Inventory (BDI) and actimetry. The results suggest that low light exposure during the day as well as higher levels of SJL are associated with depressive symptoms. The authors suggest and highlight treatment strategies aimed at alleviating circadian strain and insufficient light exposure, such as sleep education and light therapy because these measures can be very helpful in improving symptoms of depression and therefore play an important role in mental health.

## 6 Conclusion

It is not a new fact that sleep and mental health are associated and bidirectional. But what could be new and relevant is the possibility to implement more personalized items to improve mental health worldwide. Here are some ideas.

1. Measuring the Mind after Midnight by individual chronobiological analyses including the analysis of the exact circadian disruption and detecting the most prominent time of nighttime awakening that is associated with specific mental health issues such as suicidality.
2. Detecting circadian and sleep disorders by using wearables in the clinical routine and implementing sleep coaching in athletes.
3. Measuring the amount of daily light combined with the adequate implementation of light therapy as a supporting measure in the treatment of winter blues.
4. Using the data from the individual chronobiological analysis in the diagnosis of and clinical approach to treating personality disorders.
